# Ghrelin accelerates synapse formation and activity development in cultured cortical networks

**DOI:** 10.1186/1471-2202-15-49

**Published:** 2014-04-17

**Authors:** Irina I Stoyanova, Joost le Feber

**Affiliations:** 1 Biomedical Signals and Systems, Faculty of Electrical Engineering, Mathematics and Computer Sciences, Institute for Biomedical Engineering and Technical Medicine MIRA, BSS, ZH 226, University of Twente, P.O. Box 217, Enschede 7500 AE, The Netherlands; 2 Clinical Neuro Physiology, Faculty of Applied Natural Sciences, Institute for Biomedical Engineering and Technical Medicine MIRA, University of Twente, Enschede, The Netherlands

**Keywords:** Dissociated cortical neurons, Ghrelin, GHSR-1a, Electrophysiological activity, Synaptogenesis

## Abstract

**Background:**

While ghrelin was initially related to appetite stimulation and growth hormone secretion, it also has a neuroprotective effect in neurodegenerative diseases and regulates cognitive function. The cellular basis of those processes is related to synaptic efficacy and plasticity. Previous studies have shown that ghrelin not only stimulates synapse formation in cultured cortical neurons and hippocampal slices, but also alters some of the electrophysiological properties of neurons in the hypothalamus, amygdala and other subcortical areas. However, direct evidence for ghrelin’s ability to modulate the activity in cortical neurons is not available yet. In this study, we investigated the effect of acylated ghrelin on the development of the activity level and activity patterns in cortical neurons, in relation to its effect on synaptogenesis. Additionally, we quantitatively evaluated the expression of the receptor for acylated ghrelin – growth hormone secretagogue receptor-1a (GHSR-1a) during development.

**Results:**

We performed electrophysiology and immunohistochemistry on dissociated cortical cultures from neonates, treated chronically with acylated ghrelin. On average 76 ± 4.6% of the cortical neurons expressed GHSR-1a. Synapse density was found to be much higher in ghrelin treated cultures than in controls across all age groups (1, 2 or 3 weeks). In all cultures (control and ghrelin treated), network activity gradually increased until it reached a maximum after approximately 3 weeks, followed by a slight decrease towards a plateau. During early developmental stages (1–2 weeks), the activity was much higher in ghrelin treated cultures and consequently, they reached the plateau value almost a week earlier than controls.

**Conclusions:**

Acylated ghrelin leads to earlier network formation and activation in cultured cortical neuronal networks, the latter being a possibly consequence of accelerated synaptogenesis.

## Background

Ghrelin is a hormone and neuropeptide that was first identified in the stomach and initially related to the appetite stimulation and growth hormone (GH) secretion through the activation of the growth hormone secretagogue receptor-1 (GHSR-1) [1]. Ghrelin appears in two forms: unacilated ghrelin, designated as des-acyl ghrelin (DAG) and acylated ghrelin (AG), also referred to as ghrelin [2]. DAG is the dominant form in the blood plasma (90%) [3], and does not bind to the GHSR-1 [2,4]. Acylated ghrelin is produced post-translationally by octanoylation, a modification which is required for binding to the GHSR-1 [5].

The receptor has two isoforms: GHSR-1a, which, when activated by acylated ghrelin or growth hormone secretagogues (GHS), increases the intracellular Ca^2+^ concentration, and a splice variant GHSR-1b of unknown functions, which lacks the Ca^2+^ signaling capacity [6]. When the splice variant 1b is co-expressed with GHSR-1a it forms a heterodimeric association and reduces the signaling capacity of GHSR-1a [7]. GHSR-1b forms heterodimers with other G-protein-coupled receptors [8], and these heterodimeric receptors bind to peptide hormones other than ghrelin affecting intracellular signaling via activation of other pathways than Ca^2+^ signaling [9].

GHSR-1a is prominently expressed in different regions of the brain. Although the highest concentration of it is found in the pituitary gland and hypothalamus, consistent with the known role of ghrelin in GH release and the regulation of body weight and metabolism [10,11], there is also abundant GHSR-1a expression in other, extra-hypothalamic areas including the cortex and brain stem [12-17] suggesting that ghrelin plays a much broader role [10,18,19]. A recent comparative study on hippocampal and cortical dissociated neurons showed that GHSR-1 mRNA expression is higher in the hippocampus than in the cortex, and depends on the maturation stage of the cultures [20]. In humans, GHSR-1 mRNA distribution is much more widespread than that of GHSR-1a, and varies spatially and quantitatively from that of the receptor [21,22].

Soon after ghrelin’s discovery, ghrelinergic neurons were detected in different parts of the hypothalamus [23,24], including an internuclear space not involved in the metabolism regulation. This suggests a unique central role of ghrelin [25], in addition to its role as a peripheral hormone secreted by gastric endocrine glands [1]. Later on, ghrelinergic neurons have been detected also in the cerebral cortex [26-28] indicating that ghrelin might be involved in higher activities in the central nervous system, like reward, mood, learning and memory [29-32]. The central role of ghrelin has been further supported by the findings that ghrelin induces neurogenesis, as first described in the rat dorsal motor nucleus of the vagus nerve [33] and the nucleus of the solitary tract [34] and, subsequently in cultured hippocampal progenitor cells [35], the hippocampus of adult mice [36], and the rat spinal cord [37,38].

The correlation to cognitive processes was additionally confirmed by investigations on the effects of ghrelin on synapse formation and neuronal activity. In ghrelin-knockout mice ghrelin application not only increased hippocampal dendritic spine formation but also altered some of the neurophysiological properties such as long-term potentiation, leading to improved learning and memory performance [39]. Increased dendritic spine density was observed in cultured hippocampal slices when treated with ghrelin [40]. Similarly, accelerated and prolonged synaptogenesis was observed when cultured cortical neurons were chronically supplemented with ghrelin [41]. Direct application of the hormone in patch-clamp experiments has been shown to increase the electrical activity of neurons within the hypothalamic arcuate nucleus [25], the subfornical organ [42], and area postrema [43]. Long-term exposure to ghrelin in pituitary tumor cell lines also resulted in an increased frequency of spontaneous action potentials [44]. Moreover, a very recent study on the lateral amygdala provided evidence that different subpopulations of neurons display diverse responses to ghrelin [45]. However, direct evidence for acyl-ghrelin’s ability to modulate the activity in cortical neurons during the formation and development of networks is not available yet. In this study we investigated the effect of acylated ghrelin on the activity development of cortical neurons, in relation to its effect on synaptogenesis and GHSR-1a expression.

## Methods

### Dissociated cell cultures

Animal experiments were conducted according to Dutch law (as stated in the “Wet op de dierproeven”) and approved by the Utrecht Animal Use Committee (DEC). Wistar rats were bred in the local animal facility. Cortical cells were isolated from neonatal brains and plated. To obtain enough cells approximately five pups (from the same mother) were needed per plating. This approach ensured a minimum number of donor animals while obtaining sufficient experimental preparations.

Rat pups were anesthetized with isoflurane and decapitated. The brains were removed and placed in RPMI-medium (developed at Roswell Park Memorial Institute, hence the acronym RPMI). After the meninges were removed, the cortices were dissociated and collected in chemically defined R12 culture medium [46] with trypsin for further promote chemical dissociation. After the trypsin treatment, 150 μl of soybean trypsin inhibitor and 125 μl of DNAse I (20.000 units, Life Technology) were added, followed by mechanical dissociation of the neurons. The suspension was centrifuged at 1200 rpm for 5 minutes. For immunostaining, the pellet was plated on glass cover slips, while for the electrophysiological experiments, the plating was done in multi electrode arrays (MEAs) (Multi Channel Systems, Reutlingen, Germany) at a density of approximately 3000 cells/mm^2^. The electrodes of the 60 channels planar TiN/SiN MEA were 30 μm in diameter with an inter-electrode distance of 200 μm, and were arranged in a 8 *×* 8 square array (the four electrodes on the corners excluded). The impedance of the electrodes was around 200 kΩ at 1 kHz. The MEAs were pre-coated with 20 mg/ml poly-ethylene-imine (Fluka, Buchs, Switzerland) to enhance the glass-cell adhesion. In these devices, neurons form a “random” network on a global surface area of 4 mm^2^ (active surface 1*.*8 mm^2^). Cells were allowed to attach for 2 hours at 37°C and 5% CO_2_ in air and kept in R12 medium optimized with 50 ng/ml nerve growth factor (Invitrogen, Carlsbad, CA). The medium was serum-free to suppress glial cell proliferation, keeping their concentration lower than 5% [46]. The medium was renewed every 2–3 days.

### Pharmacological manipulation

Cells were kept under standard conditions of 37°C and 5% CO_2_ in air either in serum-free R12 medium as controls (*ctrl*) or with additional acylated ghrelin (*ghr*) (ab73131, Abcam, Cambridge, UK). A wide range of ghrelin concentrations ranging from 0.1 nM to 30 μM have been reported to have an excitatory, synaptogenic, proliferative and protective effect on hippocampal cells [25,35,39,43,47]. We recently showed that ghrelin accelerates synaptogenesis in a dose dependent manner using concentrations of 0.5, 1, 1.5, and 2 μM [41]. Here, we present the effect of accelerated synaptogenesis on cortical activity.

### Immunohistochemistry

We used dissociated cells from eleven plating procedures. In total 190 cultures were plated on coverslips for synaptophysin staining. About an equal number of cultures were used for each condition (*ctrl* and *ghr*). After one-, two-, or three-weeks of incubation, cultures were fixed in 4% paraformaldehyde in 0.1 M PBS, pH 7.4, and processed immunocytochemicall using the avidin-biotin-horseradish peroxidase (ABC) method [48] to detect synaptophysin. Briefly, hydrogen peroxide (0.3% in absolute methanol for 30 min) was used to inactivate endogenous peroxidase. Appropriate washes in PBS followed this and subsequent treatments. Incubation in primary antibody mouse anti-synaptophysin IgG (Abcam, Cambridge, UK, dilution 1:1000) lasted for 20 h at room temperature, followed by 2 h in biotinilated donkey anti-mouse IgG (1:500; Jackson ImmunoResearch, West) and 1 h in ABC (1:500; Vector Labs, Burlingame, CA, USA). Following rinsing, peroxidase activity was visualized using the 2.4% SG substrate kit for peroxidase (Vector) in PBS for 5 min, at room temperature. Finally, the cultures were dehydrated in a graded series of alcohols, cleared in xylene, and coverslipped with Entellan (Merck, Darmstadt, Germany). Negative controls included incubation after antigen-antibody preabsorption with the native antigen, at 4°C for 24 h, or replacement of the primary antibody with non-immune serum at the same concentration. The same procedure was applied for detection of GHSR-1a expression after one day, and one, two, three and four weeks *in vitro*, using rabbit anti-ghrelin receptor type 1a (Chemicon/Millipor, Billerica, MA, USA, dilution 1:100) as a primary antibody, biotinilated goat anti-rabbit IgG (1:500; Jackson ImmunoResearch, West) as a secondary antibody, and ABC complex (1:500; Vector Labs, Burlingame, CA, USA). Negative controls included incubation after antigen-antibody preabsorption with the native antigen, at 4°C for 24 h, or replacement of the primary antibody with non-immune serum at the same concentration.

### Data analysis and photomicrograph production

After staining, micrographs were generated at 40x and 60x using a Nikon DS-F*i*1 digital camera linked to a Nikon Eclipse 50*i* microscope. All digital images were matched for brightness in Adobe Photoshop 7.0. For quantitative analysis of synaptic marker expression, we counted the number of granules of the reaction product after synaptophysin staining. We used Nikon NIS-Elements software and obtained estimates of the mean densities and standard deviations. First, we qualitatively graded the overall density of immunostaining of neurons into three categories: high, medium and low, following the procedure described by Ljungdahl et al. [49]. Then, we calculated the granule density under high magnification at two different neurons from each category, obtained from 10 to 24 analyzed specimens per condition (*ctrl* or *ghr*) at age 1, 2 or 3 weeks. Because the largest differences in synaptic expression were detected during the first two weeks of incubation, we counted the synaptophysin expression during three weeks of incubation. To avoid bias due to differences in the cell density across the cultures, we restricted this analysis to the area of the perikarya and the initial part of the arbotizations. Analysis of neurons from all three categories yielded relatively high standard deviations in the average density per condition, per age. Two-way ANOVA was applied to assess the statistical significance of the differences in density. Known sources of variation were the treatment applied to the cultures (controls vs. ghrelin treated) and the culture age. All data were presented as mean ± standard error of the mean (SEM) unless stated otherwise. A *p*-value smaller than 0.05 was considered statistically significant.

Cell counts for GHSR-1a expression were performed on five randomly chosen cultures per age group. The fraction of GHSR-1a positive neurons was calculated per each count. All data were presented as the mean ± S.D. (standard deviation).

### Electrophysiology

Neurons were obtained from eight different plating preparations and were cultured for a period of at least four weeks in R12 supplemented with acylated ghrelin at a concentration 0.5 μM (n = 3), 1 μM (n = 3) or 2 μM (n = 5). Neuronal activity was recorded for at least one hour on at least eight different days *in vitro* (DIV). For controls, we used seven sister cultures incubated in R12. Combining the data of all experiments, we plotted the mean firing rate as a function of age. All data was grouped into bins with a bin size of five days of age. With this approach, the large differences between the activity levels of individual cultures yielded relatively large error bars.

In addition, we normalized the individual curves to their mean value before averaging across experiments. This procedure yielded curves that showed the relative development of activity, emphasizing periods of relatively fast or slow development. T-tests per age group, and two-way ANOVA were applied to assess the significance of the differences between network activity in controls and ghrelin treated cultures at different age groups. P-values <0.05 were considered as significant.

## Results

### Specificity of the immunostaining

Specificity of the immunoreaction was tested with two methods: Preincubation of the antiserums with the native proteins totally abolished the immunoreaction. Also, no labeling was observed when the antiserums were replaced by non-immune serum at the same concentration. The immunoreactivity was readily discernible at the light microscopic level by the presence of a dark-gray immunoreactive product. Neuronal structures were considered to be immunopositive when their staining was clearly stronger than that in the background.

### GHSR-1a immunoreactivity

Immunocytochemical labeling revealed that 76.3 ± 5.1% of all neurons after one day of culturing expressed GHSR-1a, unevenly distributed on the neurons. Both bipolar and multipolar neurons with several major neurites emerging from a stellate-shaped soma were GHSR-1a-immunoreactive (IR). Similar fractions of neurons expressing GHSR-1a were observed in cultures after one to four weeks (82 ± 5%, 78.2 ± 7.4%, 73.1 ± 7.4%, and 70.3 ± 10.9%, respectively). On average, 76 ± 4.6% of the neurons of all age groups exhibited GHSR-1a.

### Chronic effect of acylated ghrelin on electrical network activity and synaptophysin expression

**Figure 1:**
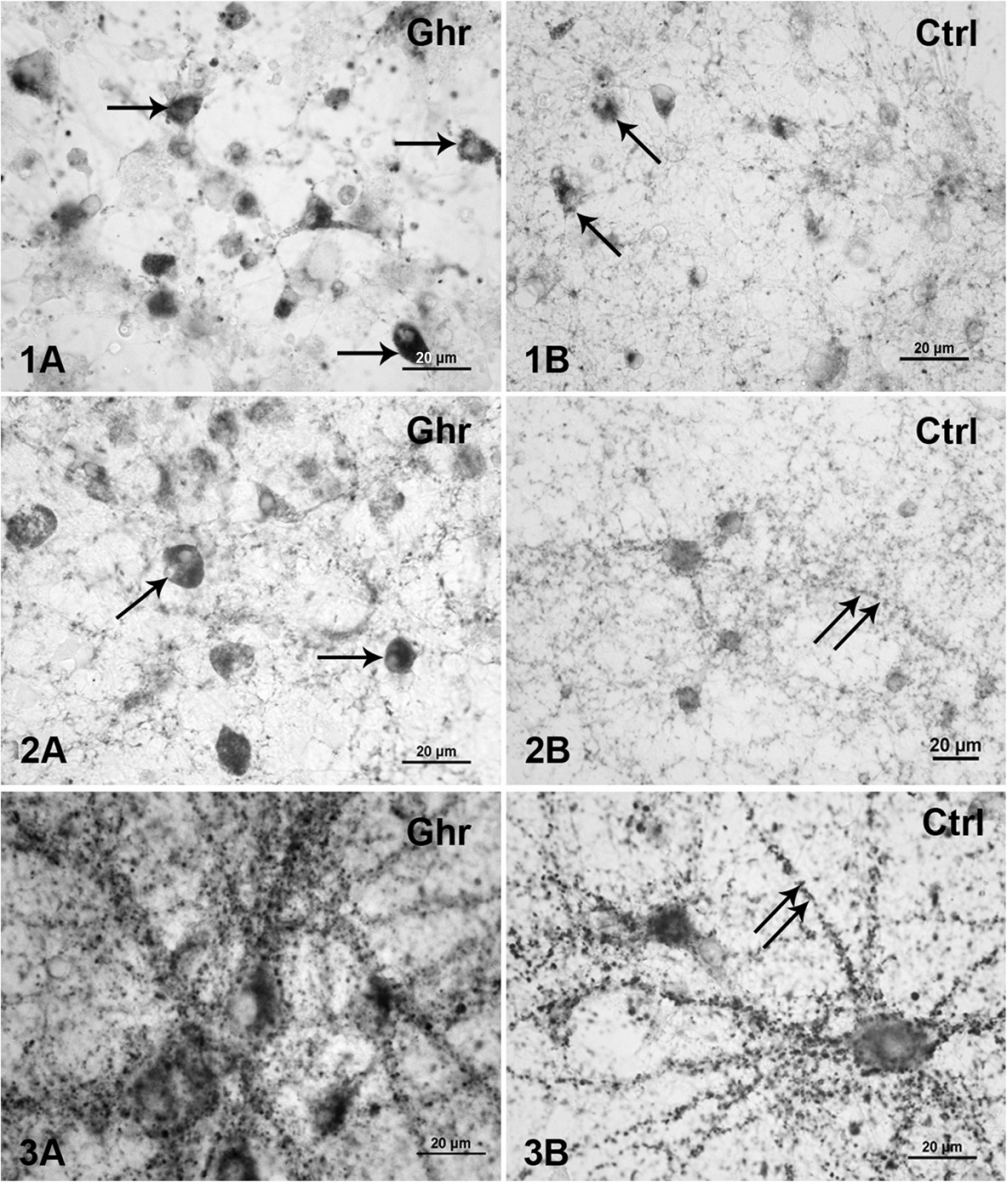
**Light microscopy of synaptophysin expression.** (1) One-week-, (2) two-weeks-, and (3) three-weeks-old neuronal cultures immunostained for synaptophysin after **(A)** 1 μM acylated ghrelin ghrelin pretreatment and **(B)** control incubation. The reaction product appeared as dark-gray dots. Arrows point at examples of neuronal perikarya with high density of staining. Double arrows point at neurites with synaptophysin expression. Scale bars: 20 μm.

**Figure 2:**
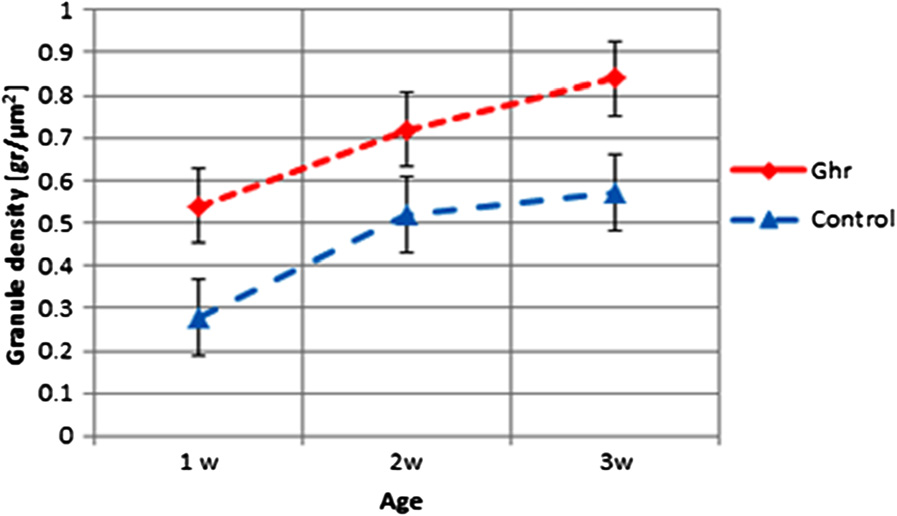
**Quantified synaptophysin expression during culture development.** Synaptophysin is quantified from images as in Figure 1 at different ages under control (*ctrl*) conditions and after chronic treatment with ghrelin (*ghr*). Expression was significantly higher in ghrelin treated cultures than in control (p < 0.001). Error bars indicate SEM.

Immunostaining for synaptophysin revealed that chronic treatment of the cultures with varying concentrations of acylated ghrelin (0.5, 1, and 2 μM) led to considerable changes in synapse development. Figure 1 shows a typical example illustrating the synapse density in cultures chronically treated with 1 μM acylated ghrelin. A week after plating in ghrelin-supplemented medium (Figure 1.1A), neurons showed some expression of synaptophysin (0.54 ± 0.13 granules/μm^2^), while in control (*ctrl)* (Figure 1.1B) it was less pronounced (0.34 ± 0.13 granules/μm^2^). Granules of reaction product were located mainly on the somata, but sparse immunoreactivity was also observed along the neurites. After two weeks *in vitro* (WIV), synaptophysin appearance was detected in all cultures (*ghr* and *ctrl*), but was far more abundant in *ghr* (0.83 ± 0.42 granules/μm^2^) (Figure 1.1A) cultures than in *ctlr* cultures (0.49 ± 0.14 granules/μm^2^) (Figure 1.1B). Both the perikarya and neurites were synaptophysin immunopositive. After three weeks of incubation we found a substantially higher expression of synaptophysin in *ghr* treated neurons (0.84 ± 0.16 granules/μm^2^) (Figure 1.3A), compared to *ctrl* (0.66 ± 0.22 granules/μm^2^) (Figure 1.3B). Two-way ANOVA showed that the synapse density significantly depended on age (p = 0.0005) and conditioning (*ghr* or *ctrl*) (p = 0.0043), but not on the interaction of these factors (p > 0.7439). The densities were always higher in *ghr* than *ctrl*. These results are illustrated in Figure 2.

**Figure 3:**
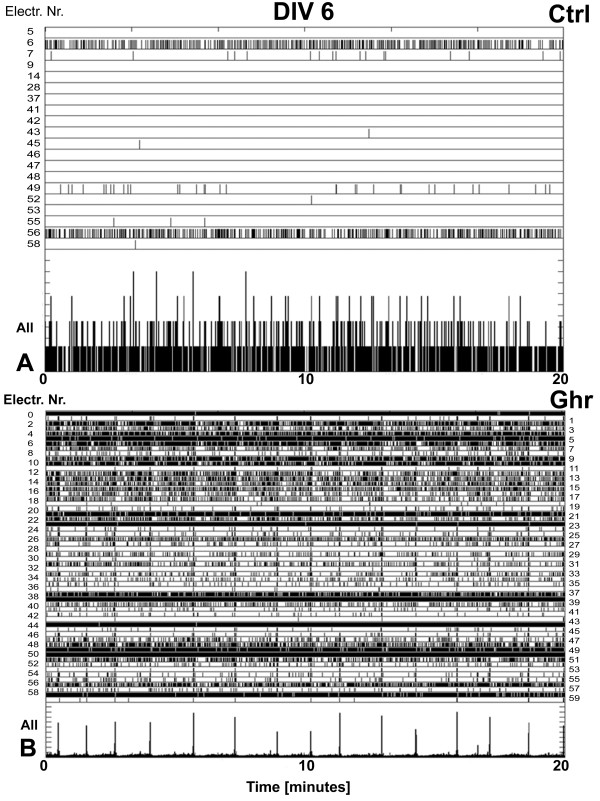
**Effect of ghrelin on network activity.** Raster plots of the neuronal activity recorded over 20 minutes (x-axis) in cultures incubated with ghrelin and controls at age DIV 6. The top rows of the panels depict only the electrodes in contact with active neurons (electrode numbers are on y-axis). Each tick represents a recorded action potential. The bottom rows of the two panels represent the summed network activity. **(A)** Network activity in a sister culture under control conditions, 47 spikes recorded in 1 minute. **(B)** Network activity in a culture chronically treated with ghrelin, 3740 spikes recorded in 1 minute.

**Figure 4:**
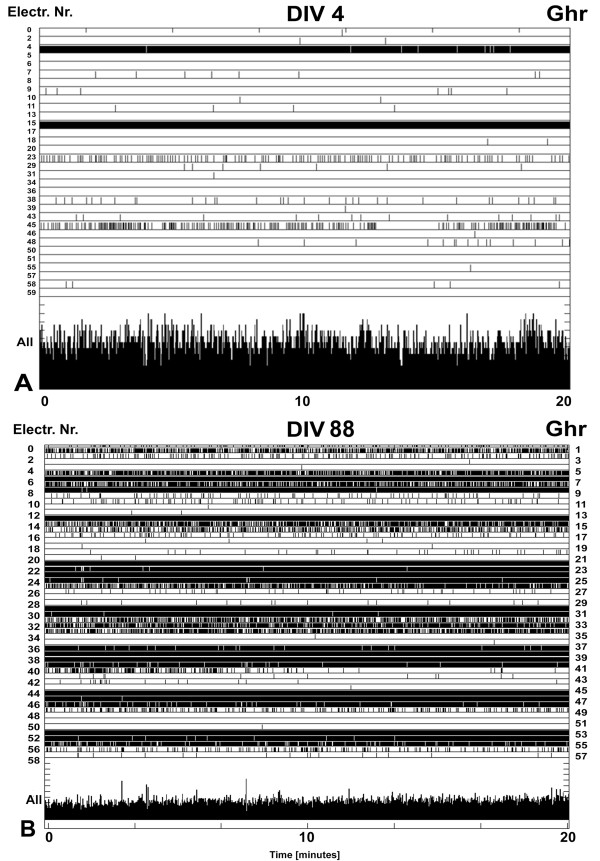
**Extensive activities recording from *ghr *culture. (A)** Raster plot of the neuronal activity recorded from ghrelin treated culture at 4 DIV (368 spikes per minute). **(B)** Raster plot of the activity recorded for 20 min from *ghr* culture at age 88 DIV. The activity level is still high (6652 spikes per minute).

Experimental data for neuronal activity were obtained from eleven ghrelin treated cultures from eight different preparations. These cultures were monitored for at least four weeks, with some of them even up to 3 months. Activity from these cultures was recorded on 12 ± 3 (SD) different days. Seven non-treated sister cultures were used as controls and all recorded for at least one hour on 10 ± 3 (SD) different days. All cultures exhibited strongly varying patterns of spontaneous activity during their temporal maturation. Initially, there was no activity but when connectivity increased some neurons became sporadically active. As network formation progressed, most neurons became active and firing patterns usually displayed network burst. When cortical networks were incubated under control conditions (Figure 3A), at 6 DIV usually only random spikes were generated at a few electrodes with no clear evidence of collective bursts. Ghrelin conditioning of the medium at all concentrations led to an earlier onset of network activity and development of bursting patterns. In general, when neurons are dissociated and cultured they show activity patterns which include periods of highly synchronized firing, behavior considered somewhat epileptic. However, we did not observe more pronounced bursting in ghrelin treated cultures except at younger culture ages, at the onset of bursting. At a young age, the activity of *ghr* cultures was substantially higher than that of the controls. In fact, in the first 10 days, the average activity was 2.3 times higher in ghrelin treated cultures. The activity pattern we observed at 6 DIV in *ghr* cultures (Figure 3B) did not normally appear before the end of the second week (11–12 DIV) in *ctrl* cultures. The *ghr* cultures exhibited some activity as early as 3–4 DIV (Figure 4A). Even after 3 months *in vitro* they still had high firing rate (Figure 4B).

**Figure 5:**
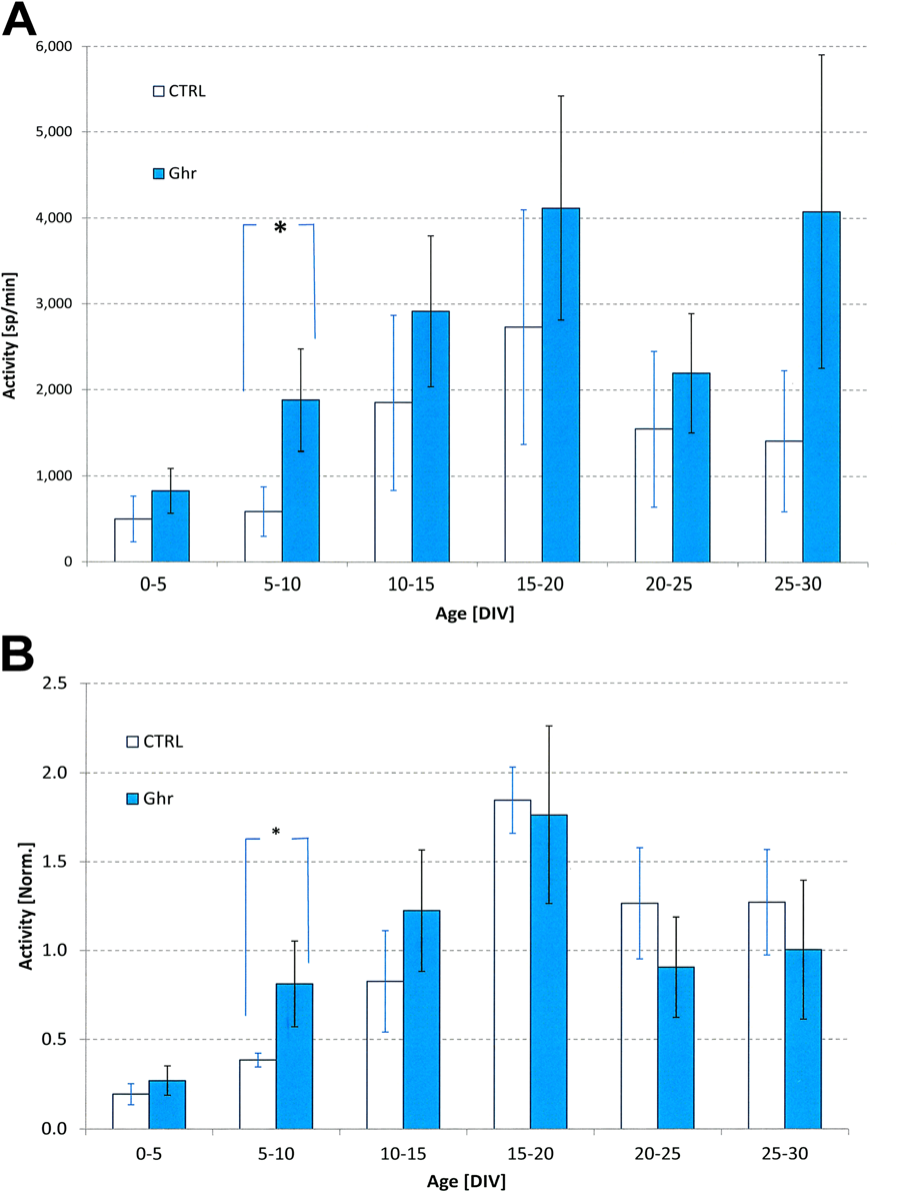
**Summary of firing rate development.** Development of the activity levels in ghrelin treated cultures (*ghr*, n = 11) and controls (ctrl, n = 7). **(A)** The average activity levels of both groups at different culture ages, up to 30 days in vitro (DIV). Error bars indicate the SEM and reflect differences between cultures. *indicates a significant difference (p < 0.04; 2-tailed t-test, unequal variances). The absolute activity levels differed substantially between cultures within either group. To emphasize the rate of development at different ages, we normalized the activity development of all individual experiments to a mean value of 1 before averaging across cultures. **(B)** The mean normalized activity levels at different ages. Error bars indicate the SEM. *indicates a significant difference (p < 0.01; 2-tailed t-test, unequal variances).

We compared the development of network activity in cultures treated with 0.5 μM (n = 3), 1 μM (n = 3), or 2 μM (n = 5) acylated ghrelin. At all concentrations, we saw increasing activity up to a maximum after 15–20 DIV, followed by a slight decrease to a plateau that lasted at least until 25–30 DIV. The absolute values of firing rates within groups showed quite large standard deviations, and differences between different concentrations were not significant at all ages (p ≥ 0.2; 2-tailed t-test, unequal variances. Mean p value of 0.52). Two way ANOVA showed that network activity significantly depended on age (p < 0.05), but not on concentration. To diminish the influence of the differences between the absolute firing rate of the individual cultures, and to focus on development, we normalized the curves of individual experiments to their mean value. Still, differences between different concentrations were not significant at all ages (p > 0.24; 2-tailed t-test, unequal variances. Mean p of 0.62). To gain statistical power, we pooled the data from all ghrelin treated cultures. Figure 5A shows the mean network activity in controls and ghrelin treated cultures at different age groups. The activity significantly depended on age (two-way ANOVA, p < 0.02) and was significantly higher in ghrelin treated cultures (two-way ANOVA, p < 0.03). Due to the large spread within groups, most differences (*ctrl* vs. *ghr*) per age group were not significant, except the difference at 5–10 DIV when *ghr* cultures were on average three times more active (p < 0.04; 2-tailed t-test, unequal variances). Then, individual curves were normalized to their mean values to emphasize periods of fast or slow development. Figure 5B shows the development of normalized network activity in cultures treated with ghrelin and control cultures. In general, in *ghr* and *ctrl* cultures the activity level initially increased until they reached their peak at 15–20 DIV and then stabilized at a slightly lower plateau. In *ghr* cultures, the mean firing rate increased much faster in young cultures up to approximately 2 weeks and first reached their plateau value before day 10, which was considerably earlier than the controls (around 15 DIV). The activity between 5 and 10 DIV was significantly higher in *ghr* than *ctrl* (p < 0.01; 2-tailed t-test, unequal variances).

## Discussion

*In vitro* cultured networks can be easily manipulated while maintaining many of the cortical cellular properties [50]. Therefore, they provide a suitable model to get a first impression of the possible effect of acylated ghrelin *in vivo*. For proper interpretation of these effects, it is important to mention that the paradigm for the natural establishment of neural circuits is known to proceed in three-stages: 1) early activity-independent wiring to produce a rough map characterized by excessive synaptic connections; 2) subsequent, use-dependent pruning to eliminate inappropriate connections and reinforce the functioning synapses [51]; and 3) succeeding homeostatic balance with reciprocal influence between the development of neuronal connectivity and intrinsic bioelectrical network activity [52].

At the end of the first phase, when neurons form extensive interconnections, they create functional networks, which exhibit frequent spontaneous action potentials discharges [53]. The frequency of these bursts of activity usually emerges towards the end of the first week *in vitro*[54], and is correlated with the age of the culture [55]. From then on the activity patterns exhibit periods of elevated firing rates with ongoing repetition of distinctive firing patterns, including network bursts, which evolve gradually in the end of the third week, followed by a drastic shortening of the network bursts after about four weeks of culturing. The burst intensity profiles become quite stable from about 30 days not only in cortical cultures [56,57], but also during the early postnatal life, as shown in lightly anesthetized rat neocortex *in vivo*[58,59]. The pattern of neuronal activity appears to be strongly dependent upon network interconnectivity [60].

Recently, ghrelin expression was found in dissociated cortical neurons with a clear conditioning- and time-related pattern in the transmitter appearance: very early ghrelin expression at a high level, followed by maturational decrease in the next two weeks of culturing [27,28]. This qualitatively mimics the *in vivo* time course of development of networks, the survival of which requires synapse consolidation and activation during the first two weeks [61,62]. In cerebral cortex cultures, the synapse density increases in parallel to the spontaneous activity development from 7 to 21 DIV [63]. In the initial stages of network development, connectivity is sparse and consequently activity is low. Still, neurons need a certain amount of activity to survive [64]. The ghrelinergic system might contribute to maintaining a healthy level of activity, possibly through accelerated synaptogenesis. Indeed, our immunostaining clearly illustrates a higher density of synapses in acylated ghrelin treated cultures than in control experiments. Because we used acylated ghrelin, which has been shown to bind only to GHSR-1a [65], and most cortical neurons did express GHSR-1a*,* the synaptogenic effect of ghrelin is most likely mediated by GHSR-1a. Acylated ghrelin increases Ca^+2^ influx [44,66]. E.g. the inositol phosphate signaling pathway, inducing intracellular calcium mobilization [44,66,67] through phospholipase C activation, is specifically associated with the GHSR-1a ligand-dependent activity [68,69]. Therefore, we hypothesize that the underlying mechanism for this synaptogenic effect of ghrelin may involve synaptic gene nuclear factors expression, many of which are calcium-dependent [70].

Ghrelin conditioning of the culturing medium resulted in earlier establishment of synaptic contacts and emerging spontaneous neuronal activity as early as 3DIV, which rapidly and continuously increased till 17–18 DIV, followed by a decline till 21–23 DIV. The first activity appeared much earlier in ghrelin treated cultures than in the controls and reached a ”mature” type (i.e. including network bursts) much earlier. In contrast, spontaneous activity of non-treated cultures usually begins toward the end of the first week *in vitro* and is initially asynchronous [54]. Thus, chronic ghrelin application had a strong stimulating effect on network activity in developing cultured cells, and to our knowledge, this study demonstrates it for the first time.

Ghrelin probably excites excitatory neurons, as well as inhibitory ones, and therefore, the total activity would increase or decrease, depending on the balance between excitation and inhibition. Approximately 20-25% of the cortical neurons are inhibitory GABAergic [71,72], and they usually express a developmental shift in their actions: at young age, GABA depolarizes postsynaptic neurons instead of hyperpolarizing them. This effect takes place during the first 8–9 postnatal days (P) or DIV and does not last beyond day 12 [73,74]. Ghrelin may directly excite those interneurons, leading to increased network activity in the first two weeks and increased inhibition after the end of the second week [75].

On the other hand, the firing rate of *ghr* cultures after three weeks *in vitro*, which is beyond this switch of GABAergic neurons, is still much higher than in *ctrl* cultures. This suggests that the predominant effect of acylated ghrelin involves the formation of excitatory synapses, thus causing augmented network activity. To validate this, it would be beneficial to determine the effect of ghrelin on postsynaptic density protein 95 (PSD-95) expression, which anchors and organizes postsynaptic neurotransmitter receptors [76] and is known to be expressed only at excitatory synapses [77,78]. However, this was beyond the scope of the current study.

The decline in firing rate observed in both *ghr* and *ctrl* cultures after three weeks, could be ascribed to the phenomenon of synaptic pruning, which is part of the natural formation of neural circuits [51], initiated by signaling through NMDA-receptors [79]. The functional significance of synapse elimination during maturation probably involves adjustment of the excitatory/inhibitory balance on individual neurons and within networks. The main argument in support of this assumption is the specificity of the loss: excitatory synapses are selectively degenerated whereas inhibitory synapses are spared [80,81]. This excitatory/inhibitory balance is important for cortical networks to function properly [82,83] and the maintenance of this balance requires a form of homeostatic plasticity. In adult cortical networks, it is dynamically regulated to avoid runaway excitation which will bring the network into an epileptic-like state or quiescence, in response to alterations in input strength [84,85]. Based on homeostatic considerations, one would expect the higher activity in cultures chronically treated with ghrelin to slow down excitatory synaptogenesis. Still, in acylated ghrelin treated cultures, we found an increased synaptogenesis and elevated firing levels, which suggests that the synaptogenic effect of ghrelin exceeded the homeostatic effect.

The stimulating effect of ghrelin during the initial stages of network development suggests that ghrelin may provide an additional mechanism to maintain healthy activity levels when activity would otherwise be too low, as may occur, for instance, after stroke. In developmental periods of low activity, synapse formation and inter-neuronal communication are both very important for brain development and functioning. Our results suggest that acylated ghrelin may support those processes, at least in our *in vitro* preparation. This is in agreement with previous studies reporting anti-appoptotic and neuroprotective effects of ghrelin [19,86,87]. It was also supported by our own findings during a difficult culturing period, when most cultures died early. Although most of those cultures became active, the duration of their activity was too short to be included in this study. Still, ghrelin treated cultures survived much longer than control cultures, which were active on average for 8 ± 7 days, while ghrelin treated cultures were active for 18 ± 6 days.

The ghrelin concentrations used in this study are relatively high, compared to physiological levels, which might induce nonspecific effects. However, it has been recently reported that *in vitro* at 1 μM concentration, 50% of the neurons of area postrema exhibit a response, 48% of the neurons react at 100 nM, 38.7% at 10 nM, and only 20% at 1 nM ghrelin [43].

Though an earlier study showed that concentration did significantly affect synapse densities [41], the results from the present experiments indicate no significant effect of different ghrelin concentrations on network activity. It is possible that activity development also depended on concentration, but our sample sizes were not large enough to detect that. However, this study did not focus on a quantitative description of dose dependent activity changes, but aimed to qualitatively describe the effect of acylated ghrelin on network activity.

## Conclusions

At all three concentrations, we observed similar differences from control activity development, supporting the conclusion that chronic acylated ghrelin application has a stimulatory effect on synaptogenesis, leads to earlier onset of activity and generation of “mature” network activity patterns, and in general to higher activity levels.

## Competing interests

The authors declare no competing interest.

## Authors’ contributions

IS and JlF have equal contribution to the study design, experiments’ performance, data analyses and manuscript preparation. IS carried out the immunohistochemical experiments and related quantification, IS and JlF carried out the electrophysiological experiments. JlF was responsible for the statistical analyses. Both authors read and approved the final manuscript.
